# IL-4 and IL-13 Receptor Signaling From 4PS to Insulin Receptor Substrate 2: There and Back Again, a Historical View

**DOI:** 10.3389/fimmu.2018.01037

**Published:** 2018-05-15

**Authors:** Achsah D. Keegan, Jose Zamorano, Aleksander Keselman, Nicola M. Heller

**Affiliations:** ^1^Department of Microbiology and Immunology, Center for Vascular and Inflammatory Diseases, University of Maryland School of Medicine, Baltimore, MD, United States; ^2^Baltimore VA Medical Center, Baltimore, MD, United States; ^3^Unidad Investigacion, Complejo Hospitalario Universitario, Caceres, Spain; ^4^Department of Anesthesiology and Critical Care Medicine, Division of Allergy and Clinical Immunology, Johns Hopkins University School of Medicine, Baltimore, MD, United States

**Keywords:** interleukin-4, interleukin-13, interleukin-4 receptor α, interleukin-13 receptor alpha1 subunit, insulin receptor substrate, IL-4-induced phosphorylated substrate, allergy, macrophage activation

## Abstract

In this historical perspective, written in honor of Dr. William E. Paul, we describe the initial discovery of one of the dominant substrates for tyrosine phosphorylation stimulated by IL-4. We further describe how this “IL-4-induced phosphorylated substrate” (4PS) was characterized as a member of the insulin receptor substrate (IRS) family of large adaptor proteins that link IL-4 and insulin receptors to activation of the phosphatidyl-inositol 3′ kinase pathway as well as other downstream signaling pathways. The relative contribution of the 4PS/IRS pathway to the early models of IL-4-induced proliferation and suppression of apoptosis are compared to our more recent understanding of the complex interplay between positive and negative regulatory pathways emanating from members of the IRS family that impact allergic responses.

## Forward by Achsah D. Keegan

Working with Dr. William E. Paul, known to all as “Bill,” was an honor and a privilege. It was also a lot of fun. In the early 1990s his laboratory was energized by studies of Th2 differentiation, the composition of the receptor for IL-4 (and later IL-13), and mechanisms of signal transduction. These studies included the identification and initial characterization of a major target for tyrosine phosphorylation in cells treated with IL-4, the focus of this perspective. As fellows, working with (not for) Bill was like being a kid in the proverbial candy shop; we were only limited by our imagination and ability to work hard. Bill’s enthusiasm for each project was infectious; he challenged all of us to think creatively and ask important questions. His scientific legacy is profound and timeless. Fascination with IL-4 signaling, starting with work in Bill’s lab, continues today; co-authors Dr. Zamorano and Dr. Heller trained as postdoctoral fellows in my laboratory before starting their own programs, and Dr. Keselman is currently a fellow in Dr. Heller’s lab. The latest research is leading to new and increasingly complex paradigms on pathway regulation with implications for the treatment of allergic diseases. And so on it goes.

## An Introduction to 4PS and the Insulin Receptor Substrate (IRS)

**Figure 1:**
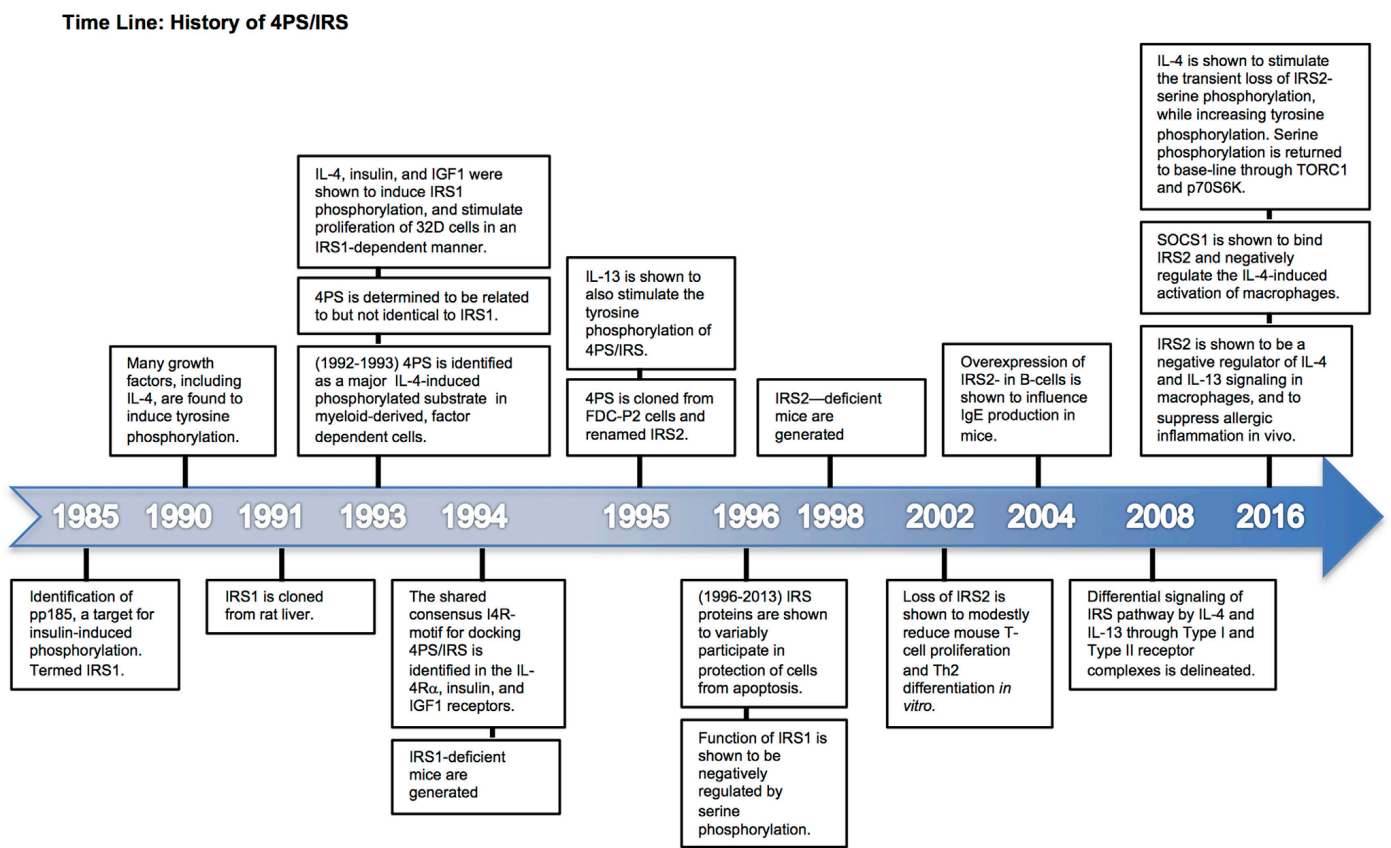
Time line of 4PS discovery and characterization. Major milestone discoveries are ordered and summarized based on the year(s) of their publication.

With the development of monoclonal antibodies capable of recognizing proteins phosphorylated on tyrosine (Y) residues, scientists were able to efficiently and consistently analyze patterns of tyrosine phosphorylation induced by a variety of growth factors and cytokines (Figure 1) (1–4). Early studies performed in collaboration with Dr. Jacalyn Pierce and Dr. Ling-Mei Wang showed that IL-4 treatment of the mouse myeloid factor-dependent cell line (FDC)-P2 stimulated the highly robust tyrosine phosphorylation of a large molecular weight protein (~180,000 Da), visible in anti-phosphotyrosine western blots of anti-phosphotyrosine precipitates, while stimulation with IL-3 failed to do so (5). We initially termed this protein IL-4-induced phosphorylated substrate or “4PS.” This phospho-protein was shown to associate with the p85 regulatory subunit of phosphatidyl-inositol (PI) 3′ kinase and with PI 3′ kinase enzyme activity.

Groups interested in the signaling pathways activated by insulin, including Dr. Kahn and Dr. White, had reported that insulin treatment of responsive cells led to the robust tyrosine phosphorylation of a large molecular weight protein (~185,000 Da) they termed insulin receptor substrate or “IRS” (6, 7). Intrigued by the similarity to 4PS, we directly compared the effects of IL-4 and insulin on tyrosine phosphorylation in FDC-P2 cells (8). Both induced the tyrosine phosphorylation of a protein with similar mobility on SDS-PAGE gels that was capable of interacting with the p85 regulatory subunit of PI 3′ kinase. Subsequent analysis of the phosphoproteins by V8 protease digestion revealed that the IL-4-induced tyrosine-phosphorylated substrate was similar to that phosphorylated in response to insulin and IGF-I suggesting that 4PS was related to IRS (8).

**Figure 2:**
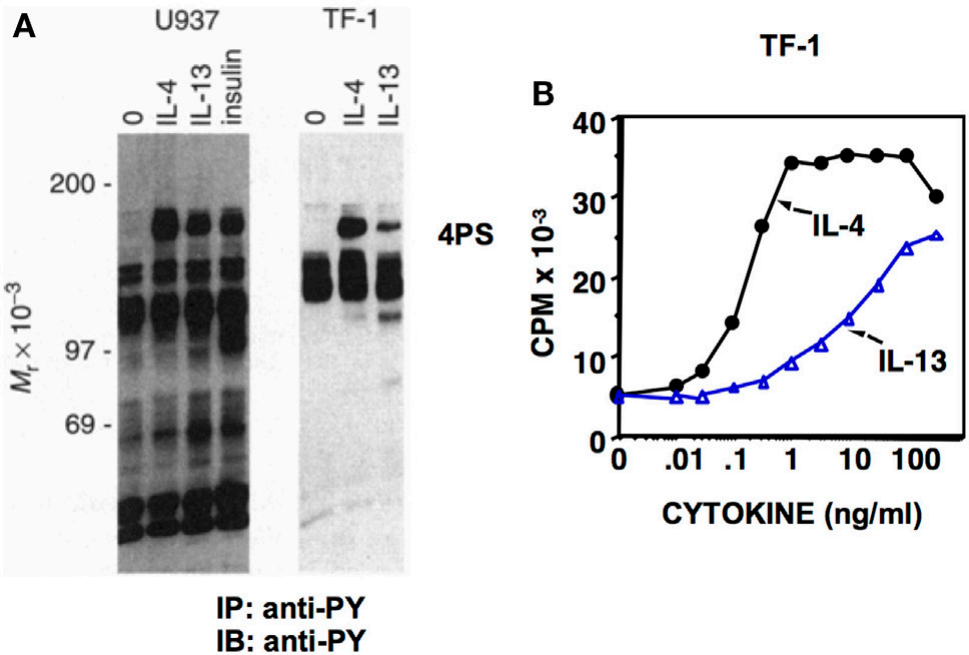
Induction of 4PS and cell proliferation by IL-4 and IL-13. **(A)** Human U937 and TF-1 cells were deprived of serum and growth factors for 2 h before treatment with human IL-4 (10 ng/ml), human IL-13 (250 ng/ml), or insulin (40 µg/ml) as indicated. Cell lysates were prepared and immunoprecipitated with the anti-phosphotyrosine antibody 4G10 followed by immunoblotting with 4G10. **(B)** TF-1 cells were incubated with the indicated doses of human IL-4 and human IL-13 for a total of 48 h. Cells were treated for the last 4 h of culture with [^3^H]thymidine. Reprinted under copyright (1995) National Academy of Sciences, U.S.A. Keegan et al. (11).

Dr. White’s group cloned the cDNA for IRS from rat liver, and it was termed IRS1 (9). The IL-3-dependent murine cell line, 32D, expressing IRS1 as a result of transfection, was generated in Dr. Pierce’s lab and used to show unequivocally that both IL-4 and insulin stimulated the tyrosine phosphorylation of IRS1 (10). This pathway was essential for the ability of IL-4 to stimulate 32D cell proliferation, and thus the concept that the 4PS/IRS pathway is required for proliferative responses was initiated. In later studies, it was observed that IL-13 also induced the tyrosine phosphorylation of 4PS (11) (Figure 2A); the potency of 4PS phosphorylation correlated with the proliferative response in human TF-1 cells (Figure 2B).

With the molecular characterization of IRS1 and development of IRS1-specific antibodies, it became clear that the 4PS protein observed in FDC-P2 cells was not IRS1 (8, 9). Polyclonal anti-IRS1 anti-serum weakly recognized 4PS in FDC lines, while two highly specific anti-IRS1 peptide antibodies were unable to precipitate 4PS. Thus, protein sequence for 4PS was obtained from anti-p85 precipitates of insulin-treated FDC-P2 cells (12). The sequence was used to generate probes to screen a cDNA library generated from FDC-P2 cells and obtain sequence for 4PS in 1995. 4PS was renamed IRS2 due to its similarity to IRS1 (12).

It is now known that IRS1 and IRS2 are members of a family of large adaptor proteins that participate in insulin, IGF-1, and IL-4 and IL-13 signaling (13). A variety of other growth factors and cytokines have also been shown to stimulate the phosphorylation of these signaling substrates (14). Both IRS1 and IRS2 can be tyrosine phosphorylated in response to IL-4 while other family members (including IRS3 or IRS4) do not appear to participate. Whether IRS1 or IRS2 or both are tyrosine phosphorylated after IL-4 stimulation depends on the cellular expression of each protein (15). Studies in 32D cells, which express neither IRS protein, revealed a positive contribution of either IRS1 or IRS2 to the IL-4-induced proliferative response (10). It was initially thought that IRS1 was predominantly expressed in non-hematopoietic cells, while IRS2 was highly expressed in cells of hematopoietic origin. However, there are exceptions to this paradigm, especially in epithelial cancers such as breast cancer (16). Furthermore, myeloid cells can express IRS1 with important functional activity as we discuss below (17). It is now appreciated that many cell types can express both family members, with differences in relative abundance that may be regulated (18).

Both IRS1 and IRS2 contain conserved amino terminal plexin homology domains and protein tyrosine binding (PTB) domains that bring these adaptors to the inner leaflet of the plasma membrane (19, 20) and interact with tyrosine-based target motifs (21), respectively. Both adaptors contain multiple tyrosines that have the potential to become phosphorylated, explaining their dominant representation in anti-phosphotyrosine immunoprecipitates. Three groups demonstrated that the Janus kinase (JAK) interacting with the cytoplasmic tail of the IL-4Rα chain, JAK1, is required for IL-4-induced tyrosine phosphorylation of IRS proteins (22–24). In collaboration with John O’Shea, we showed that IL-4 treatment lead to the activation of JAK3, while IL-13 treatment did not (11). IL-13 was shown to activate Tyk2 or in some cases JAK2 (25, 26). Both stimulated tyrosine phosphorylation of IRS2 (11).

Once phosphorylated, the tyrosine residues provide docking sites for SH2-domain-containing signaling molecules, such as the p85 subunit of PI 3′ kinase and the small protein adapter Grb2 (27). There are three tyrosines that act as p85 binding sites in IRS1 and two in IRS2 in the classic YXXM motif (13, 28). Binding of p85 to IRS proteins leads to activation of PI 3′ kinase activity and the subsequent activation of downstream signaling cascades such as the Akt pathway. The functional importance of the recruitment of the Grb2 adaptor is still unknown (27). Many other adaptor proteins have also been shown to associate with IRS1 or IRS2 including SHP-2 (also known as Syp, SH-PTP2) (29), PLC-γ (30), and SOCS proteins (31, 32), negative regulators of IL-4 signaling.

In addition to sites for tyrosine phosphorylation, both IRS1 and IRS2 have numerous potential sites for serine and threonine phosphorylation; several of these sites are unique to IRS1 and act as important modulators of functions as will be discussed in more detail in a later section (33). While well known as cytoplasmic adaptor proteins, IRS1 and IRS2 are not confined to the cytoplasm. Both can also translocate to the nucleus under certain conditions (viral/cellular transformation) and contribute to transcriptional activation or inhibition of particular genes (34–37).

## Recruitment to the IL-4 Receptor Complex: Welcome to the Insulin/IL-4 Receptor (I4R) Motif

In order to understand the mechanism by which IL-4 stimulated the tyrosine phosphorylation of 4PS/IRS and cellular proliferation, a series of deletion, mutagenesis, and pull-down studies were performed in Bill’s lab in collaboration with Dr. Keats Nelms (38). The amino acids in the cytoplasmic tail of the IL-4Rα chain responsible for 4PS/IRS binding to the human IL-4 receptor were identified between amino acids 437 and 557. Furthermore, this sequence interval was necessary for IL-4 to stimulate proliferation of 32D-IRS1 cells. Within this interval, we identified a sequence motif homologous to sites within the insulin and IGF-I receptors previously shown to bind IRS1. We named this consensus motif [^488^PL-(X)_4_-NPXYXSXSD^502^] the insulin and IL-4 Receptor (I4R) motif. The central tyrosine is critical for association of IRS proteins with the I4R motif of the IL-4Rα and for proliferation of transfected 32D-IRS1 cells (38, 39). The PTB domains of IRS1/2 recognize the core NPXY sequence when phosphorylated with influence of the amino acid residues in the −9, −8, and −7 (relative to the Y residue) positions (21, 39).

The importance of the I4R motif in dictating IL-4 receptor signaling was confirmed using domain transplant approaches (40). We generated chimeric receptors using a truncated IL-2 receptor β chain fused to the IL-4Rα domain containing the I4R motif (aa437–557) in wild type form or with the central Y residue mutated to F. Only chimeric receptors containing a wild-type I4R motif were able to mediate the tyrosine phosphorylation of IRS1 in response to IL-2.

As the IRS pathway was being characterized, contemporaneous work from several groups were on the trail of another protein tyrosine phosphorylated in response to IL-4 (41–44). This protein was identified as a member of the new (at the time) family of signal transducers and activators of transcription (STAT), and termed STAT6. The tyrosine phosphorylation of STAT6 induced by IL-4 leads to its ability to bind to STAT-palindrome sequences found in the promoters of IL-4 responsive genes such as CD23 and regulate gene transcription. While working in Bill’s lab, John Ryan showed that STAT6 was recruited to the IL-4Rα by any one of the three distinct amino acid motifs with the consensus sequence of GYKxF (45). Indeed, mutating the Y in these IL-4Rα sequences to F substantially diminished STAT6 phosphorylation in response to IL-4 and suppressed the majority of IL-4-induced responses. These STAT6 docking motifs were independent of the I4R motif. Thus, at the initial steps of signaling transduction, activation of the IRS and STAT6 pathways are independent of each other. Taken together, the published studies of the 1990s led to the conclusion that there were two major signal transduction pathways activated by IL-4. Models of the day showed that the STAT6 pathway regulated gene expression while the IRS pathway regulated cell proliferation (46–51). Later studies called this dichotomy into question as most IL-4-induced functions are greatly diminished or abrogated in STAT6-deficient mice (52–57).

## Contribution of IRS Proteins to Cell Survival

The ability of IL-4 to regulate the survival of cells is one of the important and most investigated activity of this cytokine. Soon after its characterization, IL-4 was found to exert potent anti-apoptotic activity, preventing the apoptosis of multiple cell types under different pro-apoptotic signals (58). The molecular mechanisms that signal regulation of apoptosis by IL-4 have been widely studied. These studies established that IL-4 can signal various intracellular pathways able to regulate apoptosis. Among, the molecular machinery involved in this process, the IRS proteins were found to play an important active role in the regulation of apoptosis by IL-4 (59).

As noted above, early studies performed in cell lines lacking IRS proteins demonstrated that the IL-4-induced cell proliferation was dependent on these proteins (10). Similarly, later studies performed in these cells also demonstrated a principal role of IRS proteins in the protection of apoptosis by IL-4. Thus, we showed that expression of IRS1 in 32D cells enhanced the ability of IL-4 to protect them from apoptosis after IL-3 withdrawal (59). This observation was further supported by the fact that IL-4 was not able to prevent cell death in cells expressing the Y497F mutation within the I4R motif of the IL-4Rα. This mutation abrogated the ability of IL-4 to induce IRS proteins phosphorylation. The importance of the I4R motif in regulating apoptosis was also observed in chimeric receptors consisting of a truncated form of the IL-2 receptor, unable to signal protection from apoptosis, and different fragments of the IL-4Rα (60). Transplantation of the IL-4Rα domain containing the I4R motif to the truncated IL-2 receptor transferred the ability to activate IRS proteins and to signal protection from apoptosis. This was abrogated again by the mutation Y497F within the I4R motif. These studies demonstrated the importance of the I4R motif of the IL-4Rα and the IRS proteins in the regulation of apoptosis by IL-4. In spite of these observations, the regulation of apoptosis by IL-4 seems to be more complex. IL-4 can activate IRS-independent pathways, including STAT6, to prevent cell death since IL-4 could protect from apoptosis cells lacking IRS proteins, though less effectively that in cells expressing them (59–63).

**Figure 3:**
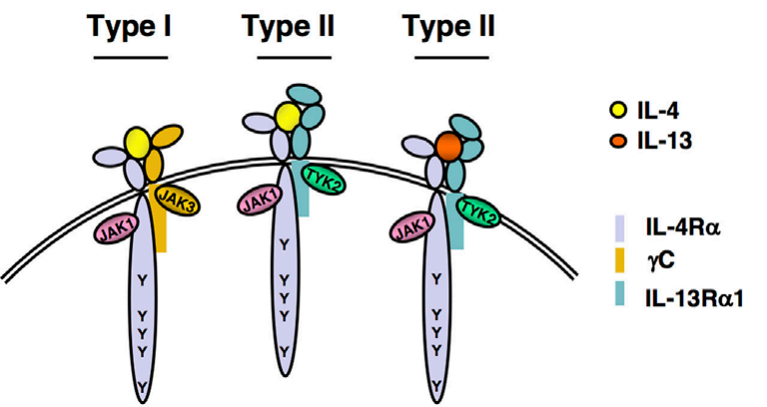
IL-4 and IL-13 receptors. A functional IL-4 receptor is composed of two transmembrane proteins. The IL-4Rα chain binds IL-4 with high affinity, leading to dimerization with the common gamma chain (γc) to form the Type I, IL-4 exclusive receptor complex or with the IL-13Rα1, to form the Type II IL-4 receptor complex. IL-13 binds to IL-13Rα1 with lower affinity, followed by heterodimerization with IL-4Rα to form the IL-13 Type II receptor complex. Following ligand binding and subunit heterodimerization, receptor-associated Janus Kinases (JAKs) become activated and phosphorylate any of the five highly conserved tyrosine residues found in the cytoplasmic tail of the IL-4Rα chain.

The IL-13 receptor complex also contains the IL-4Rα (Figure 3), sharing, therefore, intracellular molecular pathways and biological functions with IL-4 including protection from apoptosis. However, the role of IRS proteins in IL-13 signaling protection from apoptosis has not been extensively investigated. Like IL-4, IL-13 is also able to signal IRS phosphorylation (11). However, the phosphorylation of IRS2 induced by IL-13 is much weaker than by IL-4 (64). This observation could help to explain the differential described effect of IL-4 and IL-13 in apoptosis. Thus, IL-13 could reduce apoptosis in peripheral B cells although it was less potent than IL-4 (65). Both cytokines appear to activate common pathways since their effect was not additive. It may be possible that they converge on IRS2 as it has been proposed that IL-13 prevents pancreatic beta cells from apoptosis through IRS2 signaling (66).

The ability of IRS proteins to signal protection from apoptosis is not restricted to IL-4. A number of studies have shown that insulin and IGF-1 promoted pancreatic beta cell development and survival through IRS2 signaling (67). It was observed that disruption of IRS2 produced diabetes in mice by affecting development and survival of beta cells (68). By contrast, overexpression of IRS2 could improve beta cell function by protecting them from apoptosis induced by D-glucose (69). Disruption of IRS2 has been demonstrated to impair peripheral insulin signaling promoting insulin resistance in liver and skeletal muscle (68).

The effects of the IRS adaptors in preventing cell death can be extended to other cell types including hepatic, muscular, or neuronal cells. IRS2 is the main effector of insulin in the liver. IRS2 signaling has been found necessary to mediate the survival effect of insulin in neonatal hepatocytes. In this case, insulin rescue of hepatocytes from apoptosis was aborted in cells lacking IRS2 (70). The introduction of IRS2 in these cells reconstituted the ability of insulin to prevent cell death. IRS2 is overexpressed in human and murine hepatocellular carcinoma, resulting in protection from apoptosis. In these cells, downregulation of IRS2 increased apoptosis (70, 71).

Given their ability to signal protection from apoptosis, it is not surprising that IRS proteins contribute to cancer development and progression. Numerous studies have implicated IRS proteins in the progression of several tumors including breast, colorectal, prostatic, hepatic, or gastric cancers (37, 72–80). It has been proposed that IRS proteins may play an important role in breast cancer by differentially regulating cell survival, proliferation, and motility (75, 81). Increased IRS1 abundance has been associated with breast cancer cell proliferation (16). Increased IRS1 expression has been reported in primary estrogen receptor α (ERα) + breast tumors and localized breast ductal carcinoma *in situ* (37, 82). Interestingly, IRS1 interacts with ERα, and in the nucleus regulates ERα transcription (34, 36, 83–85). Furthermore, estrogen regulates expression of IRS1, thus providing a positive regulatory pathway between estrogen and the IRS1 adaptor (86). In keeping with this relationship, low IRS1 expression was observed in poorly differentiated ERα-tumors (37). On the other hand, IRS2 expression is regulated by progesterone and is associated with metastasis (81, 87, 88). The expression of IRS2 was low in ductal carcinoma *in situ* but much increased in high grade invasive human breast tumors (37). Using mouse models of breast cancer, it was shown that overexpressing IRS2 lead to mammary hyperplasia, tumorigenesis, and metastasis (74). By contrast, IRS2-deficient mammary tumor cells were less invasive and more apoptotic than cells expressing IRS2 (89, 90). Interestingly, increased expression of IRS1, but not IRS2, may favor anticancer therapies. IRS1 expression sensitized MCF-7 cells to breast cancer chemotherapeutic agents, likely by affecting Annexin-2 cellular distribution (37). Similar findings were also observed in 32D myeloid cells (91). In these cells, overexpression of IRS1, but not IRS2, also enhanced their sensitivity to chemotherapy by enhancing Annexin-A2 expression. Surprisingly, coexpression of IRS2 suppressed sensitization of chemotherapy by IRS1, and altered the subcellular localization of IRS1 and Annexin-A2 from primarily cytoplasmic to primarily nuclear. These findings suggest that analysis of the relative expression of IRS proteins may be used to predict breast cancer progression and response to chemotherapy. In this regard, other authors have proposed that IRS-specific gene expression profiles could predict the response to anti-IGF therapy in breast cancer (76).

A recent meta-analysis indicates that the IRS2 rs1805097 polymorphism can be associated with the risk of developing colorectal cancer (77). The same polymorphism has been associated with susceptibility to gastric cancer (78). In prostate, it has been reported that the IRS2/IRS1 ratio was higher in malignant compared with benign prostate tissues (79). IRS2 was also found overexpressed in human and mouse hepatocellular carcinoma cells, and down regulation of IRS2 expression increased apoptosis in these cells, suggesting that IRS2 can contribute to liver tumors (71). Furthermore, it was shown that IRS2 contributes to increased viability and reduced apoptosis in myeloid cancers harboring the activating mutation of JAK2 (JAK2V61F) by interacting with the mutant JAK2, suggesting that IRS2 can be a target to control this disease (80). These authors proposed that pharmacological inhibition of IRS2 may be useful to complement anticancer therapies by increasing apoptosis in tumor cells.

The phosphorylation of IRS proteins leads to the interaction with several signaling proteins. Among them, the PI-3′ kinase has been shown to play an important role in transmitting anti-apoptotic signals downstream of IRS2. The p85 subunit of the PI-3′ kinase coprecipitates with IRS2 and specific inhibitors of PI-3′ kinase blocked the protection from apoptosis by IL-4 on B cells (61). Similarly, the expression of dominant inhibitory forms of PI-3′ kinase abrogated the anti-apoptotic effect of IL-4 on B cells (92). Other intracellular proteins including Akt and p70S6K have been found to act downstream of IRS proteins/PI-3′ kinase in signaling protection from apoptosis (70, 93). It has also been found that insulin and IGF-1 can prevent apoptosis by an IRS2-dependent pathway that requires PI-3′ kinase and Akt (70). IRS proteins have also been reported to signal protection from apoptosis by PI-3′ kinase-independent pathways (94). Thus, the expression of IRS1, but not IRS2, protected a T cell hybridoma from activation-induced cell death (AICD) by a mechanism independent of PI-3′ kinase (94). In this case, pharmacologic inhibition of PI-3′ kinase did not abrogate the resistance of cells expressing IRS1 to AICD. In fact, the protection from apoptosis was independent of tyrosine phosphorylation and association of IRS1 with PI-3′ kinase. The authors suggested that the protection was mediated through serine residues present in IRS1 but not in IRS2. The molecular pathways activated through IRS proteins can lead to the inhibition of caspase activity (72, 73). Thus, the overexpression of IRS1 and IRS2 in neuroblastoma cells can prevent the insulin-dependent activation of caspase-3 by a PI-3′ kinase-dependent pathway (73). In the absence of IRS2, hepatocytes experience high rate of apoptosis after serum withdrawal by a mechanism involving capasase-3. Restoration of IRS2 in these cells reduced apoptosis by decreasing caspase-3 activity through a PI-3-K/Akt signaling pathway (70). In T cell hybridomas, IRS1 expression protected from apoptosis by delaying and decreasing functional FAS ligand expression after TCR engagement (94).

The fact that the IRS proteins, especially IRS2, play an important role in protection from apoptosis by several cytokines and growth factors make them potential therapeutic targets to treat several diseases. This can be useful in designing treatment strategies for certain cancers as mentioned above but also for inflammatory diseases and diabetes in which IL-4 and insulin play an important role. Several strategies to increase expression of IRS2 with pharmacologic agents are being explored to enhance pancreatic β-cell and endothelial cell survival in the context of Type II diabetes (18, 95). However, our current understanding of the relative roles of IRS1 and IRS2 in mediating and modulating allergic diseases is quite limited.

## Differential Roles of IRS2 in IL-4- Versus IL-13-Induced Allergic Responses

### IL-4 Versus IL-13—Why?

In early days, it was thought that IL-4 and IL-13 elicited identical signaling pathways (51), since they share receptor complexes (Figure 3). The Type I and Type II receptors consist of IL-4Rα/gamma chain (γ_c_) and IL-4Rα/IL-13Rα1 heterodimers, respectively (96). IL-4 binds with high affinity to the IL-4Rα inducing interaction with the γc to form a ternary complex termed the Type I receptor (Figure 3). Alternatively, the IL-4/IL-4Rα complex can interact with the IL-13Rα1 to form the Type II receptor complex. IL-13 does not bind directly to the IL-4Rα; however, its binding to the IL-13Rα1 stimulates interaction with the IL-4Rα to form a Type II receptor complex containing IL-13 instead of IL-4 (Figure 3). It is now appreciated that these three different ternary complexes activate signaling pathways that are similar but not identical to each other.

Since IL-4 has a higher affinity for initial binding to its cognate binding chain, IL-4Rα, than IL-13 has for binding to IL-13Rα1, IL-4 tends to elicit STAT6 phosphorylation at lower concentrations than IL-13 (96). However, comparisons of IL-4- and IL-13-elicited responses *in vitro* demonstrated differential biological activity on dendritic cells and macrophages (97–100). Furthermore, examination of effector functions during allergic responses in mice suggested that each cytokine controlled a different aspect of the inflammatory response. Several groups reported differences in Th2 inflammatory responses in allergic lung inflammation and worm infection models using the IL-4 and IL-13 knockout and transgenic mice (101–105). IL-4 and IL-13 were ascribed different roles in the initiation and effector phases, respectively, of allergic lung inflammation in mouse models. Only IL-4 was able to polarize T-cells to the Th2-phenotype as demonstrated by studies in IL-4-deficient (57, 101, 106) and in IL-13Rα1-deficient mice (107). This inability of IL-13 to induce Th2 polarization is easily explained by a lack of surface IL-13Rα1 expression on mouse T-cells (108, 109). The result of much research concluded that IL-4/Type I signaling elicits some of the characteristic features of allergic lung inflammation, such as eosinophilia, but that IL-13/IL-13Rα1 is required for the effector responses in the airways including airway hyperreactivity and mucus production (103–105, 107, 110).

The expression of genes characteristic of alternatively activated “M2” macrophages also demonstrated differential dependence on Type I versus Type II signaling *in vivo*. The M2 genes, *ArgI* and *Chia*, required IL-13Rα1 (Type II receptor signaling) in response to OVA challenge and intratracheal instillation of IL-13 (110). When IL-4 was intratracheally instilled into the IL-13Rα1-deficient animals, induction of *ArgI, Retnla*, and *MglI* was maintained, demonstrating that M2 responses are independent of Type II signaling *in vivo*. We also showed that M2 responses are maintained in mice lacking γc, when Th2 effectors are provided exogenously, establishing that either the Type I or Type II receptors expressed on macrophages are sufficient to drive M2 responses during allergic responses *in vivo* (111). Interestingly, Rothenberg et al. also showed that IL-13Rα1 was required for TGF-β production in response to aeroallergen challenge with *Aspergillus* or house dust mite (112). In a model of *N. brasiliensis* infection, the production of Th2 cytokines was measured in leukocytes elicited by allergic inflammation (113). Th2 cells produced both IL-4 and IL-13 in the lungs but only produced IL-4 in the lymph nodes of infected mice. Furthermore, ILC2s and basophils were major sources of IL-13, but not IL-4, in the infected lung. Although these papers described the differential production of and dependence on IL-4 and IL-13 in many of the phenotypic endpoints of Th2-mediated inflammation to allergen challenge or worm infection, few if any described the upstream signaling differences elicited by the two cytokines.

These complex modes of IL-4 and IL-13 action have great implications in the design of effective allergy therapies (114). Early attempts to suppress allergic responses in humans using a soluble form of IL-4Rα to specifically inhibit IL-4 action (it does not inhibit IL-13) did not meet clinical endpoints likely because IL-13-induced responses were not suppressed, and in addition to positive signaling pathways, IL-4 also stimulates regulatory responses that could limit inflammation, such as the suppression of TNFα production (115). Thus, it is necessary to understand the different signaling responses and downstream effects of these two cytokines to rationally design inhibitors of the IL-4- and/or IL-13-induced responses that could be used as therapeutics for asthma and allergies.

**Figure 4:**
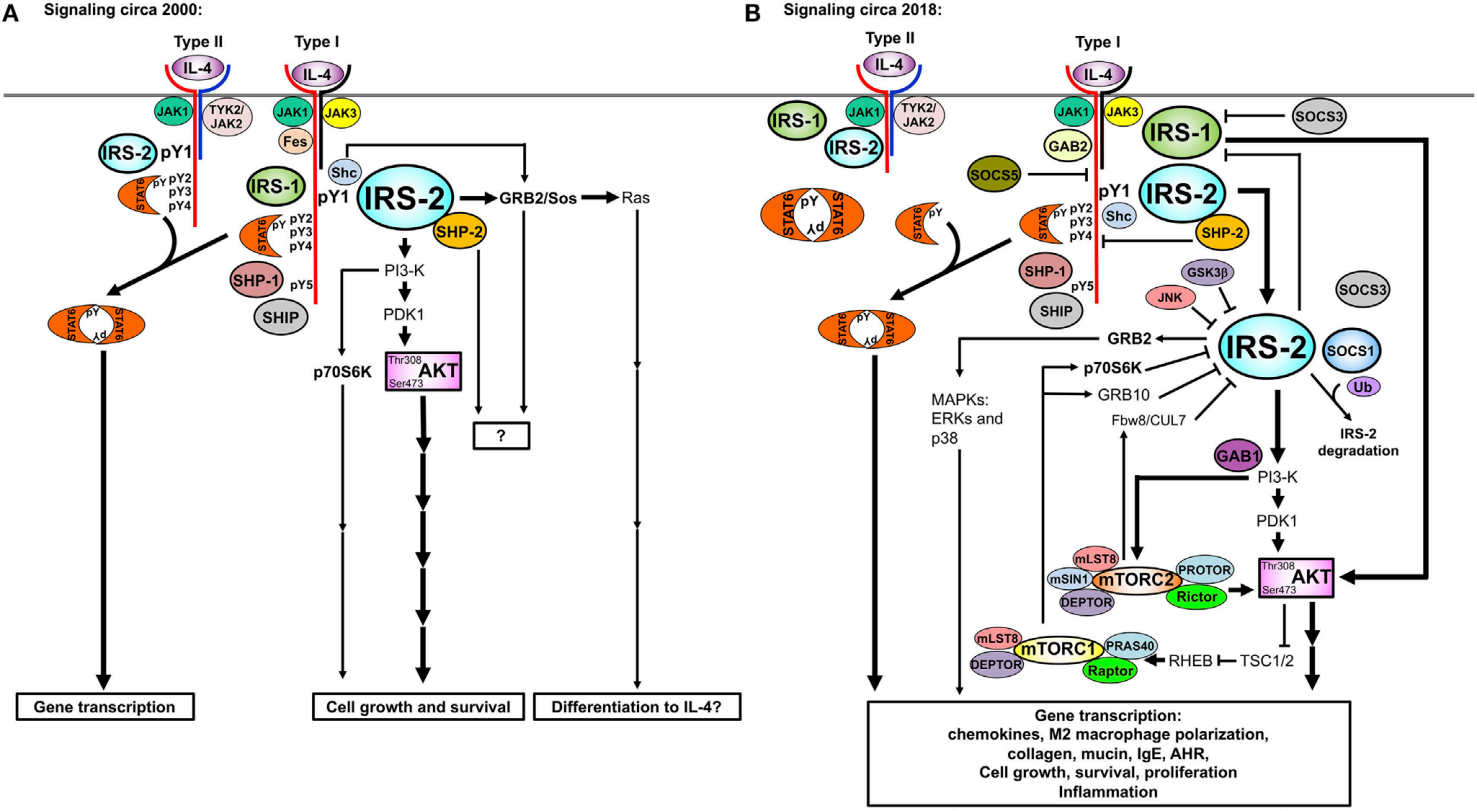
Historic **(A)** versus updated model **(B)** of IRS signaling. Our understanding of the IL-4-activated IRS signaling pathway has increased substantially between **(A)** 2000 and **(B)** 2018. The involvement of the TOR complexes, SOCS proteins, small adaptor proteins (GRBs and GABs), and other regulatory proteins in negative and positive regulation of IRS2 signaling has been uncovered. The differences in signaling triggered by IL-4 and IL-13 were determined, with IL-4 being a more potent activator of tyrosine phosphorylation of IRS2 than IL-13. Recently, IRS2 has been described as a negative regulator of IRS1, yet many interactions and roles of this pathway remain to be fully understood.

The regulation of the IRS2 pathway downstream of IL-4 signaling was described using RNAi-mediated knockdown of regulators of the TORC pathway (116). Warren et al. demonstrated that p70S6K and GRB10 were TORC1-activated negative feedback regulators of IRS2 activity (Figure 4). P70S6K regulated IL-4-induced IRS2 tyrosine phosphorylation by serine phosphorylating IRS-2. GRB10 interacted with IRS2 and with NEDD4.2 and reduced the amount of phosphorylated IRS2, likely by targeting it to the proteasome for degradation. Mice lacking TSC1/2, and the TORC1- and 2-specific proteins, Raptor and Rictor, have also been instrumental in revealing the TORC-mediated negative regulation of the IRS2 pathway (117, 118). Macrophages from Tsc1 knockout mice have increased TORC1 activity, leading to diminished Akt phosphorylation of Ser473, as might be expected when removing a negative regulator. The decrease in this surrogate measure of Akt activity resulted in decreased polarization to the M2 program in the macrophages. In terms of the role of the two TORC complexes in regulating the IL-4/IRS/Akt pathway and M2 macrophage polarization, macrophages from Rictor-deficient animals showed diminished Akt Ser 473, NDRG, and FoxO phosphorylation (117, 119, 120). Downstream, M2 polarization was either decreased (119, 120) or unchanged (117). When the TORC1 complex is inhibited with rapamycin, human monocyte-derived macrophages that are polarized to the M2 phenotype undergo apoptosis but not cells polarized to the M1 phenotype (121). Expression of M2 surface markers and other genes was reduced. We also observed a reduction in some but not all IL-4-stimulated M2 genes in a human monocytic cell line following rapamycin treatment (116).

### IL-4 Versus IL-13 Signaling Differences: IRS2

The signaling events initiated by IL-4 or IL-13 binding to their cognate receptors have largely been identified through the use of genetically altered cell lines. As discussed above, the differences in signaling between the IL-4 and IL-13 begin with the activation of different Janus family kinases (11, 24, 49, 122). The IL-4Rα associates with JAK1. The γ_c_ subunit associates with JAK3, and the IL-13Rα1 subunit associates with Tyk2 or in some cases JAK2 (Figure 3). Both receptor complexes activate STAT6 through recruitment to the IL-4Rα docking sites and tyrosine phosphorylation by the JAKs (45). Comparisons of IL-4- or IL-13-induced STAT6 phosphorylation in Ramos and A459 cells, which express either only the Type I or Type II receptor complexes, respectively, have revealed interesting differences in potency and kinetics (96). IL-4 stimulated the tyrosine phosphorylation of STAT6 faster and at lower concentrations than IL-13 in all cases, even in the absence of γ_c_ where IL-4-Type II and IL-13-Type II complexes could be compared head-to-head. Furthermore, consistent with historic studies on the exquisite sensitivity of B-cells to IL-4 (123–125), Ramos cells, expressing Type I receptors, exhibited rapid and robust STAT6 phosphorylation at low concentrations of IL-4 that was far superior to responses elicited in A549 cells (Type II receptor complex) (96). This differential responsiveness could be influenced by the relative density of the receptor chains (IL-4Rα, γc, and IL-13Rα1) and by site-directed mutagenesis of the cytokines themselves (126).

While we identified differences in potency and kinetics of STAT6 activation among the three ternary complexes, the degree of STAT6 phosphorylation could ultimately reach equality (64). However, we observed differences in activation of the IRS2 pathway that are more persistent. Comparing two monocytic cell lines, Type I and II receptor expressing U937s and Type II receptor expressing THP-1 cells, Heller et al. showed that robust tyrosine phosphorylation of IRS2 was dependent on the γ_c_ (64). Furthermore, bone marrow-derived macrophages (BMMs) lacking the γc exhibited diminished phosphorylation of IRS2 when stimulated with IL-4 while STAT6 phosphorylation was unaffected. Consistently, the Type II receptor is much less efficient at activating the IRS2 pathway even at high concentrations of cytokines that stimulate equivalent phosphorylation of STAT6.

To understand why IL-4 activates the IRS2 pathway more potently than IL-13, we dissected the role of the Type I and Type II IL-4 receptor complexes in triggering signaling. Since the IL-4Rα chain is shared between both complexes, we used human cells deficient in the γc subunit or macrophages from γc-deficient mice, as well as transfected cells expressing chimeric receptor subunits, to determine the role of the γc and IL-13Rα1 in initiating IRS2 signaling (64, 127). The presence of the γc subunit was critical for full activation of IRS2 signaling in response to IL-4 (64). However, to our surprise, it was the extracellular and transmembrane portions of the γc subunit that determined activation of the IRS2 pathway, rather than the cytoplasmic region of the γc subunit (127). We speculate that the extracellular and transmembrane regions assumed an IL-4-specific conformation that is transmitted to the associated JAKs, resulting in optimal IRS2 activation. Further research is needed to completely understand this aspect of IL-4 versus IL-13 signaling.

Once phosphorylated, IRS2 is able to associate with Grb2 and the p85 subunit of PI 3′ kinase and thereby initiates additional signaling pathways (27). We found that IL-4-activated IRS2 coprecipitates with Grb2 *via* a Type I receptor-dependent pathway (64). IL-13, while able to induce the tyrosine phosphorylation of IRS2, albeit reduced, did not stimulate the coprecipitation of IRS2 with Grb2. These results suggest that the Type I and Type II receptor complexes differentially stimulate the IRS2/Grb2 pathway. The significance of this difference is still unclear. To date, binding partners of the IRS2/Grb2 complex in the setting of IL-4 signaling have not been identified. Classical pathways downstream of Grb2 in the setting of insulin or IGF treatment such as the RAS-MAPK pathway are typically not activated by IL-4 (27, 64). Characterization of this arm of the IL-4 activated IRS2 pathway and it biological significance will require further investigation.

Activation of PI 3′ kinase through the IRS2 adaptor triggers the Akt pathway, independently of STAT6. This signal then activates the TORC1 pathway and increases the activity of downstream serine threonine kinases. Akt activation leads to the progressive degradation of TSC1/2, molecules which inhibit TORC1 and TORC2 activity (117). Enhanced TORC1 then activates GRB10 and p70 S6K. In addition to stimulating positive pathways, TORC1 induces a negative feedback loop which in the insulin signaling pathway leads to serine phosphorylation of IRS1 and reduced insulin receptor signaling (33, 128). In studies by Warren et al., it was shown that the IL-4-activated Akt/TORC1 pathway induced the serine phosphorylation of IRS2, with a decline in tyrosine-phosphorylated IRS2, indicating a reciprocal relationship between the two posttranslational modifications. Serine phosphorylation of IRS2 by p70S6K and association with GRB10 and NEDD4.2 negatively regulated IRS activity likely by targeting it for proteosomal degradation (116). Macrophages lacking TSC1 have low expression of IRS2 and fail to activate the Akt pathway when stimulated with IL-4 (117, 118). This supports the finding that TORC1 activity downmodulates IRS2 expression. SOCS1, induced during IL-4 signaling, also facilitates the ubiquitination and degradation of tyrosine-phosphorylated IRS2 to negatively regulate IRS2 signaling (32). Interestingly, a defect in SOCS1 induction was observed in allergic asthmatics, suggesting that inhibiting IRS2 signaling is protective against asthma. The proteosomal degradation of IRS1 and IRS2 is blocked by interactions with the ERα in a breast cancer cell line (36). Estrogen also enhances the expression and tyrosine phosphorylation of IRS1 in a variety of breast cancer lines (129). Whether such regulation of IRS proteins by estrogen is in some way responsible for the enhanced M2 macrophage polarization and allergic inflammatory response observed in females is not known (130, 131).

### Differential Responses on Allergic Inflammatory Cells

The difference in signaling pathways elicited by IL-4 compared to IL-13 has distinct effects on responses of cells increased in numbers during allergic responses, including M2 macrophages and eosinophils. The ability of IL-4 or IL-13 to increase expression of genes characteristic of M2 macrophage polarization correlated with the amount of tyrosine-phosphorylated IRS2 (64). On average, M2 macrophage gene expression was approximately 30–50% less in IL-13-stimulated cells compared to IL-4-stimulated cells. Even when activation of the STAT6 pathway was maximal in response to either IL-13 or IL-4, the difference in the activation of IRS2 in IL-13-stimulated compared to IL-4-stimulated BMMs resulted in less mRNA and/or protein encoding *Arg1* (Arginase 1), *Retnla* (Found in inflammatory zone 1, FIZZ1), and *Chi3l3* (YM1). Taken together, these data demonstrated that the IRS2 pathway was poorly activated in response to IL-13 and that IL-4 was a more potent inducer of the M2 macrophage polarization program than IL-13. These findings are important *in vivo* in diseases where M2 macrophages and their secreted proteins play a role in pathology or immunity, such as asthma, allergies, worm infection, or cancer. The relative presence of IL-4 or IL-13 in the microenvironment may shape the magnitude of macrophage polarization.

IL-4 and IL-13 also have distinct effects on other immune cells. IL-4 has been shown to act as a chemoattractant for human eosinophils as well as to enhance chemokine-induced movement (132). We showed that IL-4 enhanced eotaxin-1-induced chemotaxis but IL-13 did not (133). This occurred in an IL-4 concentration-dependent manner and enhancement was dependent on expression of the γc subunit and therefore Type I IL-4 receptor signaling. There were signaling differences in mouse eosinophil responses to IL-4 and IL-13. Activation of STAT6 was greater in response to IL-4 compared to IL-13. This is similar to IL-4 responses in macrophages, airway epithelial cells (A549 cell line), and other cell types. Phosphorylation of IRS2 was also greater following IL-4 stimulation but it was not statistically significant. We speculated that STAT6 signaling might synergize with eotaxin-1-/CCR3-induced PI 3′ kinaseγ activation to enhance chemotaxis through cytoskeletal rearrangement, although this remains to be elucidated. Targeting this pathway would be useful in treating Th2^hi^ allergic asthmatics, where eosinophils and Th2 cytokines play a dominant role in this asthma endotype.

## Contribution of IRS Proteins to Allergic Responses

While the STAT6 pathway has been clearly shown to be an important mediator of the majority of allergic responses *in vivo*, the contribution of the IRS pathway to immune responses is not well understood. We found that transgenic overexpression of IRS2 in lymphocytes enhanced IgE production *in vivo*, and increased the amount of IL-5 produced by *in vitro* differentiated CD4^+^ Th2 cells (134). Consistent with these findings, *in vitro* studies of T-cells isolated from IRS2^−/−^ mice found modestly reduced T-cell proliferation and production of IL-5 by Th2 cells as compared to T-cells from IRS2^+/+^ mice (62).

Mice expressing a mutation in the I4R-motif (IRS-docking site) of the murine IL-4Rα (Y500F) were developed and studied for allergic responses (135). This mutation impaired T cell proliferation but did not affect Th2 cytokine secretion *in vitro*. Surprisingly, it was found that mice expressing the Y500F form of IL-4Rα demonstrated enhanced parameters of allergic inflammation, including IgE production, airway hyperresponsiveness, eosinophilic inflammation, and mucus production, suggesting a significant contribution of this region of the IL-4Rα to inflammation control *in vivo*. While this mutation abrogated activation of the IRS2 pathway, this region of the IL-4Rα is known to act as a docking site for other signaling molecules including IRS1, Shc, FRIP1, p62DOK, and p85β (49, 136).

As discussed above, we showed that IL-4 elicited robust phosphorylation of IRS2 and M2 gene expression in macrophages *in vitro*, while IL-13 induced significantly weaker responses (64). Moreover, IL-4-mediated signaling and gene induction were reduced in macrophages lacking the γc chain and the Type I receptor. Since the PI 3′ kinase and Akt pathways downstream of IRS2 were reported to be important for M2 differentiation (137), we expected that IL-4-mediated M2 activation would be reduced in the absence of IRS2.

Contrary to expectations, stimulation of IRS2^−/−^ macrophages with either IL-4 or IL-13 enhanced expression of *Retnla, Chi3l3*, and *Arg1* mRNA, when compared to WT macrophages (17). Moreover, the differential potency of IL-4 and IL-13 for M2 gene expression was still observed in IRS2-deficient cells. Thus, the reduced quantities of M2 transcripts seen in IL-13-stimulated macrophages are not explained by reduced IRS2 phosphorylation. Another surprising finding was that loss of IRS2, an adaptor that links to PI 3′ kinase, led to increased phosphorylation of Akt and S6 under basal or IL-4-treated conditions. It is likely that this enhanced signaling proceeds *via* IRS1, as knockdown of IRS1 in the IRS2-deficient macrophages abrogated the elevated basal and IL-4-induced responses *in vitro*. These studies reveal a previously unappreciated negative feedback loop downstream of IRS2 during IL-4 signaling and suggest that the IRS1 adaptor positively regulates the M2 phenotype, although a definitive role for IRS1 remains to be established. These results are at odds with our previous work showing robust positive relationships between the IL-4-induced tyrosine phosphorylation of IRS2 and enhanced M2 macrophage differentiation (64). It is possible that compensatory mechanisms in the IRS2-deficient mice lead to enhanced involvement of the IRS1 adaptor that does not normally occur. Alternatively, it is possible that without careful analyses of the serine/threonine and tyrosine phosphorylation status of IRS1, the correlations with tyrosine-phosphorylated IRS2 are misleading (33).

Since M2 macrophages have been shown to enhance allergic responses (138–140), we further evaluated the contribution of IRS2 to allergic lung inflammation *in vivo* (17). IRS2^+/−^ and IRS2^−/−^ mice developed enhanced allergic lung inflammation and increased airway and vascular remodeling in comparison to IRS2^+/+^ mice. In the absence of IRS2, there were increased numbers of eosinophils in the airways and lungs of mice in an acute allergen sensitization/challenge model. There was also a striking increase in muscularization of small vessels that was accompanied by increased production of the M2 macrophage protein FIZZ1 by cells surrounding the blood vessels. However, there was no difference in IgE production, Th2 cytokine levels in the bronchoalveolar lavage fluid, or mucus production by airway epithelial cells. These results suggest a novel, critical role for IRS2 in limiting allergic inflammation and pulmonary arterial remodeling induced by a Th2 immune response. A potential contribution of IRS1 in allergic responses has not yet been explored in animal models or humans (141, 142).

Macrophage adoptive transfer experiments demonstrated that the negative regulation of eosinophilic inflammation and pulmonary arterial muscularization by IRS2 was at least in part intrinsic to the macrophage (17). The potential contribution of IRS1 to these *in vivo* responses is not yet known and remains an important area of investigation. These results suggest novel roles for IRS1 and IRS2 in the regulation of allergic lung disease, and present potential therapeutic strategies.

## Future Studies

The most recent work advances our understanding of the complex signaling pathways controlling allergic inflammation and paves the way for targeted manipulation of the IL-4/IL-13 pathway in the quest for additional therapeutic interventions against allergic diseases. Since the early characterization of the contribution of 4PS to cell proliferation and survival (Figure 4A), many more layers of regulation have been discovered (Figure 4B). However, the full impact of these regulatory pathways on the control of biological responses elicited by IL-4 or IL-13 are unclear. The level of complexity of potential positive and negative regulatory circuits calls for a systems engineering approach to fully understand the integration of these pathways.

Substantial progress has been made over the past 28 years in understanding the contribution of 4PS (IRS1 or IRS2) to IL-4- and IL-13-stimulated responses in the context of allergic diseases, however, as noted throughout, there is still much work to be done. Whether IRS2 is mostly a positive or a negative regulator of IL-4- or IL-13-induced responses represents a fascinating paradox. Should therapeutic strategies strive to increase or inhibit IRS2 *via* manipulation of protein expression or serine/threonine or tyrosine phosphorylation? What about IRS1? Targeting strategies are just beginning to be explored and developed in the context of epithelial cancers and Type II diabetes (18, 80, 95). What about allergic disease? On a broad and philosophical note, why does the IL-4/IL-13 system tap into the signaling pathway so critical for insulin signaling and metabolism? Do the IRS proteins play a role in the reported IL-4-induced control of adaptive thermogenesis (120, 143–145)? The search for the answers to these questions will likely engage the imagination and energy of young investigators and lead to the discovery of new and unexpected pathways controlling IL-4- and IL-13-induced responses. Bill would be delighted.

## Author Contributions

ADK developed the topic and theme of the perspective, prepared figures, wrote and edited the manuscript. JZ contributed to the writing and editing of the manuscript. NH and AK wrote and edited the manuscript and prepared Figure 4.

## Conflict of Interest Statement

The authors declare that the research was conducted in the absence of any commercial or financial relationships that could be construed as a potential conflict of interest. The reviewer HP and handling Editor declared their shared affiliation.

## References

[1] Frackelton AR Jr Ross AH Eisen HN. Characterization and use of monoclonal antibodies for isolation of phosphotyrosyl proteins from retrovirus-transformed cells and growth factor-stimulated cells. Mol Cell Biol (1983) 3:1343–52. 10.1128/MCB.3.8.1343 6194425 PMC369980

[2] Wang JY Antibodies for phosphotyrosine: analytical and preparative tool for tyrosyl-phosphorylated proteins. Anal Biochem (1988) 172:1–7. 10.1016/0003-2697(88)90403-4 2461110

[3] White MF Backer JM Preparation and use of anti-phosphotyrosine antibodies to study structure and function of insulin receptor. Methods Enzymol (1991) 201:65–79. 10.1016/0076-6879(91)01009-Q 1719349

[4] Isfort RJ Ihle JN. Multiple hematopoietic growth factors signal through tyrosine phosphorylation. Growth Factors (1990) 2:213–20. 10.3109/08977199009071507 1692720

[5] Wang L-M Keegan AD Paul WE Heidaran MA Gutkind JS Pierce JH. IL-4 activates a distinct signal transduction cascade from IL-3 in factor-dependent myeloid cells. EMBO J (1992) 12:4899–908. 1334461 10.1002/j.1460-2075.1992.tb05596.x PMC556967

[6] White MF Maron R Kahn CR. Insulin rapidly stimulates tyrosine phosphorylation of a Mr-185,000 protein in intact cells. Nature (1985) 318:183–6. 10.1038/318183a0 2414672

[7] White MF Stegmann EW Dull TJ Ullrich A Kahn CR. Characterization of an endogenous substrate of the insulin receptor in cultured cells. J Biol Chem (1987) 262:9769–77. 2439512

[8] Wang LM Keegan AD Li W Lienhard GE Pacini S Gutkind S Common elements in IL-4 and insulin signaling pathways in factor-dependent hematopoietic cells. Proc Natl Acad Sci U S A (1993) 90:4032–6. 10.1073/pnas.90.9.4032 7683417 PMC46440

[9] Sun XJ Rothenberg P Kahn CR Backer JM Araki E Wilden PA Structure of the insulin receptor substrate IRS-1 defines a unique signaling transduction protein. Nature (1991) 352:73–7. 10.1038/352073a0 1648180

[10] Wang LM Myers MG Jr Sun XJ Aaronson SA White M Pierce JH. IRS-1: essential for insulin- and IL-4-stimulated mitogenesis in hematopoietic cells. Science (1993) 261:1591–4. 10.1126/science.8372354 8372354

[11] Keegan AD Johnston J Tortolani J McReynolds LJ Kinzer CA O’Shea J Similarities and differences in signal transduction by IL-4 and IL-13: analysis of Janus kinase activation. Proc Natl Acad Sci U S A (1995) 92:7681–5. 10.1073/pnas.92.17.7681 7544000 PMC41209

[12] Sun XJ Wang LM Zhang Y Yenush L Myers MG Jr Glasheen E Role of IRS-2 in insulin and cytokine signalling. Nature (1995) 377(6545):173–7. 10.1038/377173a0 7675087

[13] White MF The IRS-signaling system: a network of docking proteins that mediate insulin and cytokine action. Recent Prog Horm Res (1998) 53:119–38. 9769706

[14] Johnston JA Wang LM Hanson EP Sun XJ White MF Oakes SA Interleukins 2, 4, 7, and 15 stimulate tyrosine phosphorylation of insulin receptor substrates 1 and 2 in T cells. Potential role of JAK kinases. J Biol Chem (1995) 270(48):28527–30. 10.1074/jbc.270.48.28527 7499365

[15] Sun XJ Pons S Wang LM Zhang Y Yenush L Burks D The IRS-2 gene on murine chromosome 8 encodes a unique signaling adapter for insulin and cytokine action. Mol Endocrinol (1997) 11(2):251–62. 10.1210/mend.11.2.9885 9013772

[16] Byron SA Horwitz KB Richer JK Lange CA Zhang X Yee D. Insulin receptor substrates mediate distinct biological responses to insulin-like growth factor receptor activation in breast cancer cells. Br J Cancer (2006) 95:1220–8. 10.1038/sj.bjc.6603354 17043687 PMC2360584

[17] Dasgupta P Dorsey NJ Li J Qi X Smith EP Keegan AD Insulin receptor substrate (IRS)-2 negatively regulates alternative macrophage activation and allergic lung inflammation. Sci Signal (2016) 9(433):ra63 10.1126/scisignal.aad6724 27330190 PMC5504879

[18] Lavan DP White MF Brazil DP. IRS proteins and diabetic complications. Diabetologia (2016) 59:2280–91. 10.1007/s00125-016-4072-7 27514532 PMC5506098

[19] Touhara K Inglese J Pitcher JA Shaw G Lefkowitz RJ. Binding of G protein beta gamma-subunits to pleckstrin homology domains. J Biol Chem (1994) 269(14):10217–20. 8144601

[20] Pitcher JA Touhara K Payne ES Lefkowitz RJ. Pleckstrin homology domain-mediated membrane association and activation of the beta-adrenergic receptor kinase requires coordinate interaction with G beta gamma subunits and lipid. J Biol Chem (1995) 270(20):11707–10. 10.1074/jbc.270.20.11707 7744811

[21] Zhou MM Huang B Olejniczak ET Meadows RP Shuker SB Miyazaki M Structural basis for IL-4 receptor phosphopeptide recognition by the IRS-1 PTB domain. Nat Struct Biol (1996) 3(4):388–93. 10.1038/nsb0496-388 8599766

[22] Wang HY Zamorano J Yoerkie J Paul WE Keegan AD IL-4-induced tyrosine phosphorylation of IRS is dependent upon expression of JAK-1 in human fibrosarcoma cells. J Immunol (1997) 158:1037–40. 9013940

[23] Burfoot MS Rogers NC Watling D Smith JM Pons S Paonessaw G Janus kinase-dependent activation of insulin receptor substrate 1 in response to interleukin-4, oncostatin M, and the interferons. J Biol Chem (1997) 272(39):24183–90. 10.1074/jbc.272.39.24183 9305869

[24] Chen XH Patel BK Wang LM Frankel M Ellmore N Flavell RA Jak1 expression is required for mediating interleukin-4-induced tyrosine phosphorylation of insulin receptor substrate and Stat6 signaling molecules. J Biol Chem (1997) 272(10):6556–60. 10.1074/jbc.272.10.6556 9045682

[25] Murata T Puri RK. Comparison of IL-13- and IL-4-induced signaling in EBV-immortalized human B cells. Cell Immunol (1997) 175(1):33–40. 10.1006/cimm.1996.1051 9015186

[26] Murata T Taguchi J Puri RK Mohri H. Sharing of receptor subunits and signal transduction pathway between the IL-4 and IL-13 receptor system. Int J Hematol (1999) 69(1):13–20. 10641437

[27] Pruett W Yuan Y Rose E Batzer AG Harada N Skolnik EY. Association between GRB2/Sos and insulin receptor substrate 1 is not sufficient for activation of extracellular signal-regulated kinases by interleukin-4: implications for Ras activation by insulin. Mol Cell Biol (1995) 15(3):1778–85. 10.1128/MCB.15.3.1778 7862167 PMC230402

[28] Sun XJ Crimmins DL Myers MG Jr Miralpeix M White MF. Pleiotropic insulin signals are engaged by multisite phosphorylation of IRS-1. Mol Cell Biol (1993) 13(12):7418–28. 10.1128/MCB.13.12.7418 7504175 PMC364813

[29] Xiao H Yin T Wang XY Uchida T Chung J White MF Specificity of interleukin-2 receptor gamma chain superfamily cytokines is mediated by insulin receptor substrate-dependent pathway. J Biol Chem (2002) 277(10):8091–8. 10.1074/jbc.M106650200 11788580

[30] Sozzani P Hasan L Séguélas MH Caput D Ferrara P Pipy B IL-13 induces tyrosine phosphorylation of phospholipase C gamma-1 following IRS-2 association in human monocytes: relationship with the inhibitory effect of IL-13 on ROI production. Biochem Biophys Res Commun (1998) 244(3):665–70. 10.1006/bbrc.1998.8314 9535722

[31] Rui L Yuan M Frantz D Shoelson S White MF. SOCS-1 and SOCS-3 block insulin signaling by ubiquitin-mediated degradation of IRS1 and IRS2. J Biol Chem (2002) 277(44):42394–8. 10.1074/jbc.C200444200 12228220

[32] McCormick SM Gowda N Fang JX Heller NM. Suppressor of cytokine signaling (SOCS)1 regulates interleukin-4 (IL-4)-activated insulin receptor substrate (IRS)-2 tyrosine phosphorylation in monocytes and macrophages via the proteasome. J Biol Chem (2016) 291(39):20574–87. 10.1074/jbc.M116.746164 27507812 PMC5034051

[33] Copps KD White MF. Regulation of insulin sensitivity by serine/threonine phosphorylation of insulin receptor substrate proteins IRS1 and IRS2. Diabetologia (2012) 55(10):2565–82. 10.1007/s00125-012-2644-8 22869320 PMC4011499

[34] Sun H Tu X Prisco M Wu A Casiburi I Baserga R. Insulin-like growth factor I receptor signaling and nuclear translocation of insulin receptor substrates 1 and 2. Mol Endocrinol (2003) 17(3):472–86. 10.1210/me.2002-0276 12554758

[35] Lassak A Del Valle L Peruzzi F Wang JY Enam S Croul S Insulin receptor substrate 1 translocation to the nucleus by the human JC virus T-antigen. J Biol Chem (2002) 277(19):17231–8. 10.1074/jbc.M110885200 11877394

[36] Morelli C Garofalo C Sisci D del Rincon S Cascio S Tu X Nuclear insulin receptor substrate 1 interacts with estrogen receptor alpha at ERE promoters. Oncogene (2004) 23(45):7517–26. 10.1038/sj.onc.1208014 15318176

[37] Porter HA Perry A Kingsley C Tran NL Keegan AD IRS-1 is highly expressed in localized breast tumors and regulates the sensitivity of breast cancer cells to chemotherapy, while IRS-2 is expressed in high grade, invasive breast tumors. Cancer Lett (2013) 338(2):239–48. 10.1016/j.canlet.2013.03.030 23562473 PMC3761875

[38] Keegan AD Nelms K White M Wang L-M Pierce JH Paul WE An IL-4 receptor region containing an insulin receptor motif is important for IL-4 mediated IRS-1 phosphorylation and proliferation. Cell (1994) 76:811–20. 10.1016/0092-8674(94)90356-5 8124718

[39] Wang HY Zamorano J Keegan AD A role for the I4R-motif of the IL-4Rα in regulating activation of the IRS2 and STAT6 pathways: analysis by mutagenesis. J Biol Chem (1998) 273:9898–905. 10.1074/jbc.273.16.9898 9545332

[40] Wang HY Paul WE Keegan AD IL-4 function can be transferred to the IL-2 receptor by tyrosine containing sequences found in the IL-4 receptor-alpha chain. Immunity (1996) 4:113–21. 10.1016/S1074-7613(00)80676-7 8624802

[41] Kotanides H Reich NC. Requirement of tyrosine phosphorylation for rapid activation of a DNA binding factor by IL-4. Science (1993) 262:1265–7. 10.1126/science.7694370 7694370

[42] Hou J Schindler U Henzel WJ Ho TC Brasseur M McKnight SL. An interleukin-4-induced transcription factor: IL-4 Stat. Science (1994) 265(5179):1701–6. 10.1126/science.8085155 8085155

[43] Schindler C Kashleva H Pernis A Rothman P. STF-IL-4: a novel IL-4-induced signal transducing factor. EMBO J (1994) 13:1350–6. 8137819 10.1002/j.1460-2075.1994.tb06388.x PMC394951

[44] Quelle FW Shimoda K Thierfelder W Fischer C Kim A Ruben SM Cloning of murine and human Stat6 (IL4-Stat): a novel stat tyrosine phosphorylated in the responses to IL-4 and IL-3 that is not required for mitogenesis. Mol Cell Biol (1995) 15:3336–43. 10.1128/MCB.15.6.3336 7760829 PMC230567

[45] Ryan JJ McReynolds LJ Wang LH Keegan AD Garfein E Rothman P IL-4-induced growth and gene expression are predominantly controlled by distinct regions of the human IL-4 receptor. Immunity (1996) 4:123–32. 10.1016/S1074-7613(00)80677-9 8624803

[46] Keegan AD Wang LM Paul WE Pierce JH Characterization of the interleukin 4 receptor. Structure and signal transduction pathways. Res Immunol (1993) 144(8):590–6. 10.1016/S0923-2494(05)80008-2 8303078

[47] Pernis A Witthuhn B Keegan AD Nelms K Garfein E Ihle JN Interleukin-4 signals through two related pathways. Proc Natl Acad Sci U S A (1995) 92:7971–5. 10.1073/pnas.92.17.7971 7544011 PMC41268

[48] Keegan AD Ryan JJ Paul WE IL-4 regulates growth and differentiation by distinct mechanisms. Immunologist (1996) 4:194–8.

[49] Nelms K Keegan AD Zamorano J Ryan JJ Paul WE. The IL-4 receptor: signaling mechanisms and biologic functions. Annu Rev Immunol (1999) 17:701–38. 10.1146/annurev.immunol.17.1.701 10358772

[50] Jian H Harris MB Rothman P IL-4/IL13 signaling beyond JAK/STAT. J Allergy Clin Immunol (2000) 105:1063–70. 10.1067/mai.2000.107604 10856136

[51] Kelly-Welch AE Hanson EM Boothby MR Keegan AD. Interleukin-4 and interleukin-13 signaling connections maps. Science (2003) 300(5625):1527–8. 10.1126/science.1085458 12791978

[52] Kaplan MH Schindler U Smiley ST Grusby MJ. Stat6 is required for mediating responses to IL-4 and for development of Th2 cells. Immunity (1996) 4(3):313–9. 10.1016/S1074-7613(00)80439-2 8624821

[53] Takeda K Tanaka T Shi W Matsumoto M Minami M Kashiwamura S Essential role of Stat6 in IL-4 signalling. Nature (1996) 380(6575):627–30. 10.1038/380627a0 8602263

[54] Shimoda K van Deursen J Sangster MY Sarawar SR Carson RT Tripp RA Lack of IL-4-induced Th2 response and IgE class switching in mice with disrupted Stat6 gene. Nature (1996) 380(6575):630–3. 10.1038/380630a0 8602264

[55] Kuperman D Schofield B Wills-Karp M Grusby MJ. Signal transducer and activator of transcription factor 6 (Stat6)-deficient mice are protected from antigen-induced airway hyperresponsiveness and mucus production. J Exp Med (1998) 187(6):939–48. 10.1084/jem.187.6.939 9500796 PMC2212182

[56] Chapoval SP Dasgupta P Smith EP DeTolla LJ Lipsky MM Kelly-Welch AE STAT6 expression in multiple cell types mediates the cooperative development of allergic airway disease. J Immunol (2011) 186(4):2571–83. 10.4049/jimmunol.1002567 21242523 PMC3139332

[57] Dasgupta P Chapoval SP Smith EP Keegan AD. Transfer of in vivo primed transgenic T cells supports allergic lung inflammation and FIZZ1 and Ym1 production in an IL-4Rα and STAT6 dependent manner. BMC Immunol (2011) 20(12):60. 10.1186/1471-2172-12-60 22014099 PMC3212823

[58] Paul WE Interleukin-4: a prototypic immunoregulatory lymphokine. Blood (1991) 77:1859–70. 2018830

[59] Zamorano J Wang HY Wang LM Pierce JH Keegan AD. IL-4 protects cells from apoptosis via the insulin receptor substrate pathway and a second independent signaling pathway. J Immunol (1996) 157:4926–34. 8943397

[60] Zamorano J Keegan AD Regulation of apoptosis by tyrosine-containing domains of the IL-4Rα: Y497 and Y713, but not the STAT6-docking tyrosines, signal protection from apoptosis. J Immunol (1998) 161:859–67. 9670964

[61] Zamorano J Kelly AE Austrian J Wang HY Keegan AD Costimulation of resting B lymphocytes alters the IL-4-activated IRS-2 signaling pathway in a STAT6 independent manner: implications for cell survival and proliferation. Cell Res (2001) 11:44–54. 10.1038/sj.cr.7290065 11305324

[62] Wurster AL Withers DJ Uchida T White MF Grusby MJ. Stat6 and IRS-2 cooperate in interleukin 4 (IL-4)-induced proliferation and differentiation but are dispensable for IL-4-dependent rescue from apoptosis. Mol Cell Biol (2002) 22:117–26. 10.1128/MCB.22.1.117-126.2002 11739727 PMC134231

[63] Wurster AL Rodgers VL White MF Rothstein TL Grusby MJ. Interleukin-4-mediated protection of primary B cells from apoptosis through Stat6-dependent up-regulation of Bcl-xL. J Biol Chem (2002) 277(30):27169–75. 10.1074/jbc.M201207200 12023955

[64] Heller NM Qi X Juntilla I Shirey KA Vogel SN Paul WE Type I IL-4 receptors selectively activate IRS-2 to induce target gene expression in macrophages. Sci Signal (2008) 1(51):ra17 10.1126/scisignal.1164795 19109239 PMC2739727

[65] Lømo J Blomhoff HK Jacobsen SE Krajewski S Reed JC Smeland EB. Interleukin-13 in combination with CD40 ligand potently inhibits apoptosis in human B lymphocytes: upregulation of Bcl-xL and Mcl-1. Blood (1997) 89:4415–24. 9192766

[66] Rütti S Howald C Arous C Dermitzakis E Halban PA Bouzakri K. IL-13 improves beta-cell survival and protects against IL-1beta-induced beta-cell death. Mol Metab (2015) 5:122–31. 10.1016/j.molmet.2015.11.003 26909320 PMC4735661

[67] Withers DJ Burks DJ Towery HH Altamuro SL Flint CL White MF. IRS-2 coordinates IGF-1 receptor-mediated beta-cell development and peripheral insulin signalling. Nat Genet (1999) 23:32–40. 10.1038/12631 10471495

[68] Withers DJ Gutierrez JS Towery H Burks DJ Ren JM Previs S Disruption of IRS-2 causes type 2 diabetes in mice. Nature (1998) 391:900–4. 10.1038/36116 9495343

[69] Mohanty S Spinas GA Maedler K Zuellig RA Lehmann R Donath MY Overexpression of IRS2 in isolated pancreatic islets causes proliferation and protects human beta-cells from hyperglycemia-induced apoptosis. Exp Cell Res (2005) 303:68–78. 10.1016/j.yexcr.2004.09.011 15572028

[70] Valverde AM Fabregat I Burks DJ White MF Benito M. IRS-2 mediates the antiapoptotic effect of insulin in neonatal hepatocytes. Hepatology (2004) 40:1285–94. 10.1002/hep.20485 15565601

[71] Boissan M Beurel E Wendum D Rey C Lécluse Y Housset C Overexpression of insulin receptor substrate-2 in human and murine hepatocellular carcinoma. Am J Pathol (2005) 167:869–77. 10.1016/S0002-9440(10)62058-5 16127164 PMC1698721

[72] Kim B Feldman EL. Insulin receptor substrate (IRS)-2, not IRS-1, protects human neuroblastoma cells against apoptosis. Apoptosis (2009) 14:665–73. 10.1007/s10495-009-0331-0 19259821 PMC5499040

[73] Stöhr O Hahn J Moll L Leeser U Freude S Bernard C Insulin receptor substrate-1 and -2 mediate resistance to glucose-induced caspase-3 activation in human neuroblastoma cells. Biochim Biophys Acta (2011) 1812:573–80. 10.1016/j.bbadis.2011.02.006 21354306

[74] Dearth RK Cui X Kim HJ Kuiatse I Lawrence NA Zhang X Mammary tumorigenesis and metastasis caused by overexpression of insulin receptor substrate 1 (IRS-1) or IRS-2. Mol Cell Biol (2006) 26:9302–14. 10.1128/MCB.00260-06 17030631 PMC1698542

[75] Nagle JA Ma Z Byrne MA White MF Shaw LM. Involvement of insulin receptor substrate 2 in mammary tumor metastasis. Mol Cell Biol (2004) 24:9726–35. 10.1128/MCB.24.22.9726-9735.2004 15509777 PMC525494

[76] Becker MA Ibrahim YH Oh AS Fagan DH Byron SA Sarver AL Insulin receptor substrate adaptor proteins mediate prognostic gene expression profiles in breast cancer. PLoS One (2016) 11:e0150564. 10.1371/journal.pone.0150564 26991655 PMC4798554

[77] Yin J Zhang Z Zheng H Xu L. IRS-2 rs1805097 polymorphism is associated with the decreased risk of colorectal cancer. Oncotarget (2017) 8:25107–14. 10.18632/oncotarget.15342 28212577 PMC5421913

[78] Zhao XM Chen J Yang L Luo X Xu LL Liu DX Association between IRS-2 G1057D polymorphism and risk of gastric cancer. World J Gastrointest Oncol (2012) 4:9–15. 10.4251/wjgo.v4.i1.9 22347534 PMC3277875

[79] Heni M Hennenlotter J Scharpf M Lutz SZ Schwentner C Todenhöfer T Insulin receptor isoforms A and B as well as insulin receptor substrates-1 and -2 are differentially expressed in prostate cancer. PLoS One (2012) 7:e50953 10.1371/journal.pone.0050953 23251408 PMC3519512

[80] de Melo Campos P Machado-Neto JA Eide CA Savage SL Scopim-Ribeiro R da Silva Souza Duarte A IRS2 silencing increases apoptosis and potentiates the effects of ruxolitinib in JAK2V617F-positive myeloproliferative neoplasms. Oncotarget (2016) 7:6948–59. 10.18632/oncotarget.6851 26755644 PMC4872760

[81] Gibson SL Ma Z Shaw LM. Divergent roles for IRS-1 and IRS-2 in breast cancer metastasis. Cell Cycle (2007) 6:631–7. 10.4161/cc.6.6.3987 17361103

[82] Sisci D Morelli C Garofalo C Romeo F Morabito L Casaburi F Expression of nuclear insulin receptor substrate 1 in breast cancer. J Clin Pathol (2007) 60:633–41. 10.1136/jcp.2006.039107 16882697 PMC1955087

[83] Jackson JG White MF Yee D. Insulin receptor substrate-1 is the predominant signaling molecule activated by insulin-like growth factor-I, insulin, and interleukin-4 in estrogen receptor-positive human breast cancer cells. J Biol Chem (1998) 273:9994–10003. 10.1074/jbc.273.16.9994 9545345

[84] Chen J Wu A Sun H Drakas R Garofalo C Cascio S Functional significance of type 1 insulin-like growth factor-mediated nuclear translocation of the insulin receptor substrate-1 and beta-catenin. J Biol Chem (2005) 280:29912–20. 10.1074/jbc.M504516200 15967802

[85] Tu X Batta P Innocent N Prisco M Casaburi I Belletti B Nuclear translocation of insulin receptor substrate-1 by oncogenes and Igf-I. Effect on ribosomal RNA synthesis. J Biol Chem (2002) 277:44357–65. 10.1074/jbc.M208001200 12202493

[86] Molloy CA May FE Westley BR. Insulin receptor substrate-1 expression is regulated by estrogen in the MCF-7 human breast cancer cell line. J Biol Chem (2000) 275:12565–71. 10.1074/jbc.275.17.12565 10777546

[87] Cui X Lazard Z Zhang P Hopp TA Lee AV. Progesterone crosstalks with insulin-like growth factor signaling in breast cancer cells via induction of insulin receptor substrate-2. Oncogene (2003) 22:6937–41. 10.1038/sj.onc.1206803 14534541

[88] Vassen L Wegrzyn W Klein-Hitpass L. Human insulin receptor substrate-2 (IRS-2) is a primary progesterone response gene. Mol Endocrinol (1999) 13:485–94. 10.1210/me.13.3.485 10077005

[89] Jackson JG Zhang X Yoneda T Yee D. Regulation of breast cancer cell motility by insulin receptor substrate-2 (IRS-2) in metastatic variants of human breast cancer cell lines. Oncogene (2001) 20:7318–25. 10.1038/sj.onc.1204920 11704861

[90] Ma Z Gibson SL Byrne MA Zhang J White MF Shaw LM. Suppression of insulin receptor substrate 1 (IRS-1) promotes mammary tumor metastasis. Mol Cell Biol (2006) 26:9338–51. 10.1128/MCB.01032-06 17030605 PMC1698550

[91] Porter HA Carey GC Keegan AD Insulin receptor substrate (IRS) 1 expression enhances the sensitivity of 32D cells to chemotherapy-induced cell death. Exp Cell Res (2012) 318(14):1745–58. 10.1016/j.yexcr.2012.04.020 22652453 PMC3395425

[92] Carey GB Semenova E Qi X Keegan AD IL-4 protects the B-cell lymphoma cell line CH31 from anti-IgM-induced growth arrest and apoptosis: contribution of the PI-3’kinase/Akt pathway. Cell Res (2007) 17:942–55. 10.1038/sj.cr.2007.90 17968425

[93] Lin SJ Chang C Ng AK Wang SH Li JJ Hu CP. Prevention of TGF-beta-induced apoptosis by interlukin-4 through Akt activation and p70S6K survival signaling pathways. Apoptosis (2007) 12:1659–70. 10.1007/s10495-007-0085-5 17624592

[94] Li L Qi X Williams M Shi Y Keegan AD Overexpression of IRS1, but not IRS2, protects a T-cell hybridoma from activation-induced cell death (AICD). J Immunol (2002) 168:6215–23. 10.4049/jimmunol.168.12.6215 12055235

[95] Kuznetsova A Yu Y Hollister-Lock J Apare-Addo L Rozzo A Sadagurski M Trimeprazine increases IRS2 in human islets and promotes pancreatic β cell growth and function in mice. JCI Insight (2016) 1(3):e80749 10.1172/jci.insight.80749 27152363 PMC4854304

[96] LaPorte SL Juo JS Vaclavikova J Colf L Qi X Heller NM Molecular basis of cytokine receptor pleiotropy in the Interleukin-4/13 system. Cell (2008) 132:259–72. 10.1016/j.cell.2007.12.030 18243101 PMC2265076

[97] Zhang MZ Wang X Wang Y Niu A Wang S Zou C IL-4/IL-13-mediated polarization of renal macrophages/dendritic cells to an M2a phenotype is essential for recovery from acute kidney injury. Kidney Int (2017) 91(2):375–86. 10.1016/j.kint.2016.08.020 27745702 PMC5548101

[98] Jones LH Cook PC Ivens AC Thomas GD Phythian-Adams AT Allen JE Modulation of dendritic cell alternative activation and function by the vitamin A metabolite retinoic acid. Int Immunol (2015) 27(11):589–96. 10.1093/intimm/dxv020 25899567 PMC4625886

[99] Bhattacharjee A Shukla M Yakubenko VP Mulya A Kundu S Cathcart MK. IL-4 and IL-13 employ discrete signaling pathways for target gene expression in alternatively activated monocytes/macrophages. Free Radic Biol Med (2013) 54:1–16. 10.1016/j.freeradbiomed.2012.10.553 23124025 PMC3534796

[100] Cook PC Jones LH Jenkins SJ Wynn TA Allen JE MacDonald AS. Alternatively activated dendritic cells regulate CD4+ T-cell polarization in vitro and in vivo. Proc Natl Acad Sci U S A (2012) 109(25):9977–82. 10.1073/pnas.1121231109 22660926 PMC3382483

[101] Coyle AJ Le Gros G Bertrand C Tsuyuki S Heusser CH Kopf M Interleukin-4 is required for the induction of lung Th2 mucosal immunity. Am J Respir Cell Mol Biol (1995) 13(1):54–9. 10.1165/ajrcmb.13.1.7598937 7598937

[102] Rankin JA Picarella DE Geba GP Temann UA Prasad B DiCosmo B Phenotypic and physiologic characterization of transgenic mice expressing interleukin 4 in the lung: lymphocytic and eosinophilic inflammation without airway hyperreactivity. Proc Natl Acad Sci U S A (1996) 93(15):7821–5. 10.1073/pnas.93.15.7821 8755560 PMC38832

[103] Wills-Karp M Luyimbazi J Xu X Schofield B Neben TY Karp CL Interleukin-13: central mediator of allergic asthma. Science (1998) 282(5397):2258–61. 10.1126/science.282.5397.2258 9856949

[104] Grunig G Warnock M Wakil AE Venkayya R Brombacher F Rennick DM Requirement for IL-13 independently of IL-4 in experimental asthma. Science (1998) 282(5397):2261–3. 10.1126/science.282.5397.2261 9856950 PMC3897229

[105] Zhu Z Homer RJ Wang Z Chen Q Geba GP Wang J Pulmonary expression of interleukin-13 causes inflammation, mucus hypersecretion, subepithelial fibrosis, physiologic abnormalities, and eotaxin production. J Clin Invest (1999) 103(6):779–88. 10.1172/JCI5909 10079098 PMC408149

[106] Gavett SH O’Hearn DJ Karp CL Patel EA Schofield BH Finkelman FD Interleukin-4 receptor blockade prevents airway responses induced by antigen challenge in mice. Am J Physiol (1997) 272(2 Pt 1):L253–61. 9124376 10.1152/ajplung.1997.272.2.L253

[107] Munitz A Brandt EB Mingler M Finkelman FD Rothenberg ME. Distinct roles for IL-13 and IL-4 via IL-13 receptor alpha1 and the type II IL-4 receptor in asthma pathogenesis. Proc Natl Acad Sci U S A (2008) 105(20):7240–5. 10.1073/pnas.0802465105 18480254 PMC2386078

[108] Walter DM McIntire JJ Berry G McKenzie AN Donaldson DD DeKruyff RH Critical role for IL-13 in the development of allergen-induced airway hyperreactivity. J Immunol (2001) 167(8):4668–75. 10.4049/jimmunol.167.8.4668 11591797

[109] Nono JK Ndlovu H Abdel Aziz N Mpotje T Hlaka L Brombacher F. Interleukin-4 receptor alpha is still required after Th2 polarization for the maintenance and the recall of protective immunity to Nematode infection. PLoS Negl Trop Dis (2017) 11(6):e0005675. 10.1371/journal.pntd.0005675 28651009 PMC5501681

[110] Ramalingam TR Pesce JT Sheikh F Cheever AW Mentink-Kane MM Wilson MS Unique functions of the type II interleukin 4 receptor identified in mice lacking the interleukin 13 receptor alpha1 chain. Nat Immunol (2008) 9(1):25–33. 10.1038/ni1544 18066066 PMC2692551

[111] Dasgupta P Qi X Smith EP Keegan AD Absence of the common gamma chain (γC), a critical component of the type I IL-4 receptor, increases the severity of allergic lung inflammation driven by adoptively transferred TH2 cells. PLoS One (2013) 8(8):e71344 10.1371/journal.pone.0071344 23940740 PMC3734063

[112] Rothenberg ME Wen T Shik D Cole ET Mingler MM Munitz A IL-13 receptor {alpha}1 differentially regulates aeroallergen-induced lung responses. J Immunol (2011) 187(9):4873–80. 10.4049/jimmunol.1004159 21957151 PMC3197875

[113] Liang HE Reinhardt RL Bando JK Sullivan BM Ho IC Locksley RM. Divergent expression patterns of IL-4 and IL-13 define unique functions in allergic immunity. Nat Immunol (2011) 13(1):58–66. 10.1038/ni.2182 22138715 PMC3242938

[114] Sheridan C Drugmakers cling to dual IL-13/4 blockbuster hopes. Nat Biotechnol (2018) 36:3–5. 10.1038/nbt0118-3 29319691

[115] Steinke JW Negri J Enelow R Baramki DF Borish L Proinflammatory effects of IL-4 antagonism. J Allergy Clin Immunol (2006) 118(3):756–8. 10.1016/j.jaci.2006.05.002 16950298

[116] Warren KJ Fang X Gowda NM Thompson JJ Heller NM. The TORC1-activated proteins, p70S6K and GRB10, regulate IL-4 signaling and M2 macrophage polarization by modulating phosphorylation of insulin receptor substrate-2. J Biol Chem (2016) 291(48):24922–30. 10.1074/jbc.M116.756791 27742835 PMC5122764

[117] Byles V Covarrubias AJ Ben-Sahra I Lamming DW Sabatini DM Manning BD The TSC-mTOR pathway regulates macrophage polarization. Nat Commun (2013) 4:2834. 10.1038/ncomms3834 24280772 PMC3876736

[118] Zhu L Yang T Li L Sun L Hou Y Hum X TSC1 controls macrophage polarization to prevent inflammatory disease. Nat Commun (2014) 5:4696. 10.1038/ncomms5696 25175012

[119] Festuccia WT Pouliot P Bakan I Sabatini DM Laplante M. Myeloid-specific Rictor deletion induces M1 macrophage polarization and potentiates in vivo pro-inflammatory response to lipopolysaccharide. PLoS One (2014) 9(4):e95432. 10.1371/journal.pone.0095432 24740015 PMC3989321

[120] Hallowell RW Collins SL Craig JM Zhang Y Oh M Illei PB mTORC2 signalling regulates M2 macrophage differentiation in response to helminth infection and adaptive thermogenesis. Nat Commun (2017) 8:14208. 10.1038/ncomms14208 28128208 PMC5290163

[121] Mercalli A Calavita I Dugnani E Citro A Cantarelli E Nano R Rapamycin unbalances the polarization of human macrophages to M1. Immunology (2013) 140(2):179–90. 10.1111/imm.12126 23710834 PMC3784164

[122] Oakes SA Candotti F Johnston JA Chen YQ Ryan JJ Taylor N Signaling via IL-2 and IL-4 in JAK3-deficient severe combined immunodeficiency lymphocytes: JAK3-dependent and independent pathways. Immunity (1996) 5(6):605–15. 10.1016/S1074-7613(00)80274-5 8986719

[123] Howard M Farrar J Hilfiker M Johnson B Takatsu K Hamaoka T Identification of a T cell-derived B cell growth factor distinct from interleukin 2. J Exp Med (1982) 155:914–23. 10.1084/jem.155.3.914 6977612 PMC2186613

[124] Farrar JJ Howard M Fuller-Farrar J Paul WE. Biochemical and physicochemical characterization of mouse B cell growth factor: a lymphokine distinct from interleukin 2. J Immunol (1983) 131(4):1838–42. 6352806

[125] Howard M Paul WE Interleukins for B lymphocytes. Lymphokine Res (1982) 1(1):1–4. 6985399

[126] Junttila IS Creusot RJ Moraga I Bates DL Wong MT Alonso MN Redirecting cell-type specific cytokine responses with engineered interleukin-4 superkines. Nat Chem Biol (2012) 8(12):990–8. 10.1038/nchembio.1096 23103943 PMC3508151

[127] Heller NM Qi X Gesbert F Keegan AD The extracellular and transmembrane domains of the γc and IL-13Rα1 chains, not their cytoplasmic domains, dictate the nature of signaling responses to IL-4 and IL-13. J Biol Chem (2012) 287(38):31948–61. 10.1074/jbc.M112.348896 22829596 PMC3442527

[128] Carlson CJ White MF Rondinone CM. Mammalian target of rapamycin regulates IRS-1 serine 307 phosphorylation. Biochem Biophys Res Commun (2004) 316(2):533–9. 10.1016/j.bbrc.2004.02.082 15020250

[129] Lee AV Jackson JG Gooch JL Hilsenbeck SG Coronado-Heinsohn E Osborne CK Enhancement of insulin-like growth factor signaling in human breast cancer: estrogen regulation of insulin receptor substrate-1 expression in vitro and in vivo. Mol Endocrinol (1999) 13(5):787–96. 10.1210/mend.13.5.0274 10319328

[130] Melgert BN Postma DS. All men are created equal? New leads in explaining sex differences in adult asthma. Proc Am Thorac Soc (2009) 6(8):724–7. 10.1513/pats.200906-054DP 20008884

[131] Keselman A Fang X White PB Heller NM. Estrogen signaling contributes to sex differences in macrophage polarization during asthma. J Immunol (2017) 199:1573–83. 10.4049/jimmunol.1601975 28760880 PMC5576568

[132] Dubois GR Schweizer RC Versluis C Bruijnzeel-Koomen CA Bruijnzeel PL. Human eosinophils constitutively express a functional interleukin-4 receptor: interleukin-4-induced priming of chemotactic responses and induction of PI-3 kinase activity. Am J Respir Cell Mol Biol (1998) 9(4):691–9. 10.1165/ajrcmb.19.4.3208 9761767

[133] Heller NM Gwinn WM Constant SL Keegan AD IL-4 engagement of the type I IL-4 receptor complex enhances murine eosinophil migration to eotaxin-1 in vitro. PLoS One (2012) 7(6):e39673 10.1371/journal.pone.0039673 22761864 PMC3386270

[134] Kelly-Welch AE Wang HY Wang L-M Pierce JH Jay G Finkelman F Transgenic expression of IRS2 in murine B-cells alters the cell-density dependence of IgE production in vitro and enhances IgE production in vivo. J Immunol (2004) 172:2803–10. 10.4049/jimmunol.172.5.2803 14978080

[135] Blaeser F Bryce PJ Ho N Raman V Dedeoglu F Donaldson DD Targeted inactivation of the IL-4 receptor alpha chain I4R motif promotes allergic airway inflammation. J Exp Med (2003) 198(8):1189–200. 10.1084/jem.20030471 14557412 PMC2194235

[136] Nelms K Snow AL Hu-Li J Paul WE. FRIP, a hematopoietic cell-specific rasGAP-interacting protein phosphorylated in response to cytokine stimulation. Immunity (1998) 9(1):13–24. 10.1016/S1074-7613(00)80584-1 9697832

[137] Weisser SB McLarren KW Voglmaier N van Netten-Thomas CJ Antov A Flavell RA Alternative activation of macrophages by IL-4 requires SHIP degradation. Eur J Immunol (2011) 41(6):1742–53. 10.1002/eji.201041105 21469115 PMC6902421

[138] Kim EY Battaile JT Patel AC You Y Agapov E Grayson MH Persistent activation of an innate immune response translates respiratory viral infection into chronic lung disease. Nat Med (2008) 14(6):633–40. 10.1038/nm1770 18488036 PMC2575848

[139] Ford AQ Dasgupta P Mikhailenko I Smith EP Noben-Trauth N Keegan AD Adoptive transfer of IL-4Rα^+^ macrophages is sufficient to support Th2-driven alternative macrophage activation and to enhance eosinophilic inflammation in a mouse model of allergic lung inflammation. BMC Immunol (2012) 13:6 10.1186/1471-2172-13-6 22292924 PMC3283450

[140] Dasgupta P Keegan AD. Contribution of alternatively activated macrophages to allergic lung inflammation: a tale of mice and men. J Innate Immun (2012) 4(5–6):478–88. 10.1159/000336025 22440980 PMC6741623

[141] Tamemoto H Kadowaki T Tobe K Yagi T Sakura H Hayakawa T Insulin resistance and growth retardation in mice lacking insulin receptor substrate-1. Nature (1994) 372:182–6. 10.1038/372182a0 7969452

[142] Araki E Lipes MA Patti ME Bruning JC Haag B III Johnson RS Alternative pathway of insulin signaling in mice with targeted disruption of the IRS-1 gene. Nature (1994) 372:186–90. 10.1038/372710b0 7526222

[143] Wu D Molofsky AB Liang HE Ricardo-Gonzalez RR Jouihan HA Bando JK Eosinophils sustain adipose alternatively activated macrophages associated with glucose homeostasis. Science (2011) 332(6026):243–7. 10.1126/science.1201475 21436399 PMC3144160

[144] Qiu Y Nguyen KD Odegaard JI Cui X Tian X Locksley RM Eosinophils and type 2 cytokine signaling in macrophages orchestrate development of functional beige fat. Cell (2014) 157:1292–308. 10.1016/j.cell.2014.03.066 24906148 PMC4129510

[145] Rao RR Long JZ White JP Svensson KJ Lou J Lokurkar I Meteorin-like is a hormone that regulates immune-adipose interactions to increase beige fat thermogenesis. Cell (2014) 157(6):1279–91. 10.1016/j.cell.2014.03.065 24906147 PMC4131287

